# Association between different metabolic phenotypes of obesity and thyroid disorders among Chinese adults: a nationwide cross-sectional study

**DOI:** 10.3389/fendo.2023.1158013

**Published:** 2023-04-21

**Authors:** Bo Song, Cihang Lu, Di Teng, Zhongyan Shan, Weiping Teng

**Affiliations:** Department of Endocrinology and Metabolism, Institute of Endocrinology, National Health Commission Key Laboratory of Diagnosis and Treatment of Thyroid Diseases, The First Hospital of China Medical University, Shenyang, Liaoning, China

**Keywords:** metabolic health, obesity, TIDE, thyroid disorders, prevalence

## Abstract

**Background:**

Increased body mass index (BMI) and metabolic abnormalities both have potential associations with thyroid disease. The aim of this study was to investigate the correlation between different metabolic phenotypes of obesity and thyroid disorders using nationwide data from China.

**Methods:**

Data were collected from a cross-sectional survey called the Thyroid Disorders, Iodine Status, and Diabetes Epidemiological Survey conducted between 2015 and 2017 in China. A total of 69007 subjects aged 18 years or older were defined and divided into six groups on the basis of BMI and metabolic health status: metabolically healthy normal weight (MHNW), metabolically unhealthy normal weight (MUNW), metabolically healthy overweight (MHOW), metabolically unhealthy overweight (MUOW), metabolically healthy obesity (MHO), and metabolically unhealthy obesity (MUO). We estimated the odds ratios (ORs) and confidence intervals [CIs] for different thyroid disorders according to metabolic phenotypes using multivariate logistic regression models.

**Results:**

In our study, we found that the prevalence of subclinical hypothyroidism was almost as high in the MUNW group as in the MUO group, the prevalence of overt hyperthyroidism was highest in the MUNW group and Graves’ disease was highest in the MHO group. Our results also suggested that the prevalence of goiter and thyroid nodules increased with increased BMI values and that the MUO group had the highest incidence. Multivariate logistic regression analysis indicated that subjects with unhealthy metabolic phenotypes (MUNW, MUOW, and MUO) all had an increased risk of subclinical hypothyroidism, regardless of their BMI. MUNW subjects had an approximately 1.6-fold higher risk of overt hyperthyroidism and a 1.8-fold higher risk of Graves’ disease than their metabolically healthy counterparts (MHNW). The present study also demonstrated that the MUO group had the highest risk of goiter and thyroid nodules among the metabolic phenotypes of obesity.

**Conclusion:**

Based on our study, we found that metabolic abnormalities and obesity play different roles in various thyroid diseases. Metabolically unhealthy individuals, both with and without obesity, have a higher risk of thyroid disorders than metabolically healthy individuals without obesity.

## 1 Introduction

With rapid economic development and lifestyle changes, obesity has become a common health problem in both developed and developing countries (1, 2). The prevalence of obesity has increased rapidly worldwide. Ward et al. (3) suggested that up to 57.8% of the population of the United States will be overweight or obese by 2030. Due to its high incidence worldwide, it is essential to find a more scientific treatment for obesity that may help save health care resources. Metabolic health is also receiving increasing attention. Recent studies identified a subgroup of obese subjects with a low burden of metabolic abnormalities as having the so-called metabolically healthy obesity (MHO) phenotype (4). Metabolic health refers to the absence of both metabolic syndrome and any known diabetes, coronary artery disease, stroke, hypertension or dyslipidemia (5). In addition, not all individuals in the normal weight range present with a healthy metabolic and disease-free profile. Approximately 20% of the normal weight adult population is metabolically unhealthy, displaying an increased risk of obesity-related abnormalities such as reduced insulin sensitivity, elevated blood pressure, atherogenic lipids, and cardiovascular events (6, 7).

In recent years, several studies have shown that metabolic syndrome (MetS) and obesity are independent risk factors for developing serious diseases, such as type 2 diabetes mellitus (T2DM) (8), cardiovascular disease (CVD) (9), and malignant tumors (10). However, few studies have explored the relationship between metabolic obesity phenotypes and thyroid disorders. The thyroid plays an important role in metabolic regulation. Thyroid hormones have multiple effects on glucose and lipid metabolism, blood pressure regulation, and energy consumption. Recent studies found that patients with hypothyroidism had an increased risk of metabolic syndrome (11). Sari et al. discovered that functional and morphological alterations of the thyroid gland were associated with obesity (12). In addition, increased thyroid volume and nodule prevalence were reported for the first time insulin resistance(IR) patients in an iodine-sufficient area (13).

Thyroid Disorders, Iodine Status and Diabetes (TIDE), a national epidemiological cross-sectional study, was conducted from 2015 to 2017 covering all 31 provinces of mainland China. The overall results have been previously reported (14). All subjects received a complete questionnaire survey, physical examination, laboratory examination, and body composition examination. Baseline data for body mass index (BMI), metabolic status and thyroid function were collected to identify the association between the metabolic phenotype and thyroid disorders in the Chinese population. Therefore, the present study aimed to investigate this association between metabolic phenotypes and thyroid disorders using TIDE data.

## 2 Materials and methods

### 2.1 Subjects

For this cross-sectional study, we obtained data from subjects who participated in the TIDE survey. The TIDE survey is a national epidemiological cross-sectional survey conducted in 31 provincial regions of mainland China using a whole-cluster, stratified random sampling design. The specific sampling and implementation methods have been previously published and described in **Supplementary Method 1** (15). A total of 80937 adults were deemed eligible for this study and completed the survey. In the current analysis, we excluded 6467 subjects who did not have demographic and thyroid function test information as well as 5463 individuals who had incomplete data regarding metabolic status. Therefore, the final sample consisted of 69007 respondents. The flowchart of patient inclusion is shown in **Supplementary Figure S1**. This study was conducted according to the Declaration of Helsinki as revised in 2013, and the protocol was approved by the Ethics Committee of China Medical University (2014-103-2; serial number: IRB [2008]115). All participants gave their written informed consent before this study.

### 2.2 Data collection

Basic sociodemographic and clinical information about the subjects was obtained by trained investigators using a standardized questionnaire. This information included age, sex, education level, regional location, personal and family medical history, lifestyle habits such as current smoking status, family income, and household salt consumption. Standardized measurements of the participants’ height, weight, waist circumference (WC), and blood pressure (BP) were taken. BMI was calculated by dividing weight in kg by height in square meters (kg/m2). Prior to measuring BP, participants were asked to rest in a seated position for 5 minutes, and three measurements were taken using a mercury sphygmomanometer. The average of three readings was calculated for systolic and diastolic BP. Height, weight, and waist and hip circumference were measured with the participant in light clothing and without shoes.

All participants underwent an oral glucose tolerance test (OGTT) after 10 hours of overnight fasting followed by early morning fasting blood and spot urine sample collection. These samples were used for basic biochemical examinations, including fasting plasma glucose (FPG), lipid profile, glycosylated hemoglobin (HbA1c), thyroid function, 2-hour plasma glucose (2-hPG), uric acid (UA), and urinary iodine concentration (UIC). Serum and urine samples were obtained by centrifugation of the blood samples and preserved at -20°C before analysis. Upon completion of the survey and specimen collection, all specimens were transported *via* a cold chain system to the central laboratory in Shenyang, China.

### 2.3 Laboratory measurements

Total cholesterol (TC), high-density lipoprotein cholesterol (HDL-C), triglycerides (TGs) and low-density lipoprotein cholesterol (LDL-C) were measured with an automatic biochemical analyzer (Mindray BS-180 Analyzer). Bio-Rad reagents were used for HbA1c measurement. We used the hexokinase enzymatic method to measure fasting blood glucose. Serum thyroid-stimulating hormone (TSH) and free thyroxine (FT4) values were measured with an electrochemiluminescence immunoassay on a Cobas 601 analyzer (Roche Diagnostics, Switzerland). Serum TSH, thyroid peroxidase antibodies (TPOAbs) and thyroglobulin antibodies (TGAbs) were measured with an electrochemiluminescence immunoassay on a Cobas 601 analyzer (Roche Diagnostics, Switzerland). When the TSH level exceeded the upper limit of the reference range (0.27-4.20 mIU/L), FT4 and free triiodothyronine (FT3) levels were measured. UIC was measured by inductively coupled plasma−mass spectrometry (Agilent 7700x; Agilent Technologies, Santa Clara, CA). All participants underwent thyroid ultrasonography by specially trained technicians using a portable instrument (LOGIQ 100 PRO; GE, Milwaukee, WI, with 7.5-MHz linear transducers). Two trained quality control personnel apart from the sonographers were responsible for supervising the accuracy and reliability of the ultrasound results.

### 2.4 Definition of body weight status and metabolic phenotype

A definition for metabolically healthy adults has recently been proposed that meets all of the following criteria (1): systolic blood pressure (SBP) ≤130 mmHg, diastolic blood pressure (DBP) ≤85 mmHg, no antihypertensive treatment and no self-reported history of hypertension; (2) HDL-cholesterol>1 mmol/L (>40 mg/dl) for men and >1.3 mmol/l (>50 mg/dl) for women; (3) serum triglycerides ≤ 1.7 mmol/l (≤150 mg/dl); and (4) fasting plasma glucose ≤ 5.6 mmol/l (≤100 mg/dl) and no drug treatment with glucose-lowering agents and no self-reported history of diabetes (16). Body weight status was categorized according to the Working Group on Obesity in China (17) as normal weight (BMI ≥ 18.5 and < 24 kg/m2), overweight (BMI≥ 24 and < 28 kg/m2), and obesity (BMI≥28 kg/m2). In addition, the metabolically unhealthy phenotype was defined within each BMI group who did not meet at least one of the four criteria. Based on the BMI categories and metabolic health status, all study subjects were divided into 6 groups: metabolically healthy normal weight (MHNW), metabolically healthy overweight (MHOW), metabolically healthy obesity (MHO), metabolically unhealthy normal weight (MUNW), metabolically unhealthy overweight (MUOW), and metabolically unhealthy obesity (MUO).

### 2.5 Clinical diagnosis

The diagnostic criteria for thyroid disorders are listed in **Table 1**.

**Table 1:** Diagnostic criteria for thyroid disorders.

	Thyroid Disorders	Diagnostic Criteria
1	Overt hyperthyroidism	TSH <0.27 mIU/L; fT4 > 22 pmol/L or fT3 > 6.8 pmol/L
2	Subclinical hyperthyroidism	TSH <0.27 mIU/L; fT4 and fT3 within the normal range (fT4 12.0–22.0 pmol/L; fT3 3.1–6.8 pmol/L)
3	Graves’ disease	Overt hyperthyroidism or subclinical hyperthyroidism; TRAb >1.75 IU/L or a diffuse goiter on B-mode ultrasonography
4	Overt hypothyroidism	TSH >4.2 mIU/L; fT4 < 12 pmol/L
5	Subclinical hypothyroidism	TSH >4.2 mIU/L; fT4 within 12–22 pmol/L
6	AIT	TPOAbs > 34 IU/ml or TgAbs > 115 IU/ml
7	Goiter for adults	Female >22.5 mL; male >25.4 mL
8	Thyroid nodule	One or more nodule (>5 mm) without goiter

fT3, free triiodothyronine; fT4, free thyroxine; TgAb, thyroglobulin antibodies; TPOAb, thyroid peroxidase antibodies; TRAb, TSH receptor antibodies; TSH, thyrotropin.

### 2.6 Statistical analysis

All statistical analyses were performed using SPSS software (version 23.0; IBM Corp., Armonk, NY, USA). Statistical significance was defined as P<0.05. To obtain national estimates, all calculations were weighted to represent the overall Chinese adult population aged 18 years or older using the weighted coefficients derived from 2010 China population census data and the sampling scheme of the current survey. Standard errors were calculated with appropriate statistical techniques with data from the complex survey design. We performed a descriptive analysis of BMI, UIC, TPOAbs, TgAbs, TSH levels, and thyroid volume using quartile stratification and compared the medians for differences using Student’s independent t test. To assess the independence of two categorical variables, the chi-square test or Fisher’s exact test was used. Baseline characteristics are presented as weighted means ± standard errors (SEs) for continuous variables and numbers (percentages) for categorical variables depending on their type. Univariable and multivariable logistic regression were used to estimate odds ratios (ORs) with 95% confidence intervals [CIs] for the association between the six metabolic obesity categories and the risk of thyroid disorders. We fitted 3 logistic regression models. Model 1 was a crude model that was not adjusted for covariates. Model 2 was adjusted for age, sex and ethnicity. Model 3 was further adjusted for smoking status, educational level, family income, UIC, TPOAbs, and TgAbs except for AIT, which was adjusted for smoking status, educational level, family income and UIC. In all analyses, P < 0.05 was considered statistically significant.

## 3 Results

### 3.1 Baseline characteristics of the participants

**Table 2** summarizes the demographic and biochemical characteristics of the six groups of participants, which included 69007 subjects (34690 men and 34317 women). In the study population, the metabolically unhealthy group was the largest group (64.10%). Among the normal weight participants, almost half were metabolically unhealthy (MHNW 26.14%, MUNW 25.47%). Among the rest of the participants, with the increase in BMI, the proportion of metabolically unhealthy individuals gradually increased (MHOW 8.00%, MUOW 26.00%, MHO 1.75%, MUO 12.54%). Compared with the metabolically healthy group, metabolically unhealthy subjects were more likely to be older and male (*P*< 0.0001). The metabolically unhealthy group had more current smokers, more rural residents, lower educational levels and lower family incomes than the metabolically healthy group. In addition, people of Han ethnicity were more likely to be metabolically unhealthy than people of other ethnicities. Compared with metabolically healthy individuals, the metabolically unhealthy population comprised individuals with higher anthropometric indexes (BMI, WC), individuals with a more unfavorable risk profile (higher levels of blood pressure, FPG, uric acid, TSH and HbA1c, dyslipidemia, and lower levels of HDL-C) and individuals with lower UIC values (*P*< 0.0001). In addition, within the different metabolic status groups, the above indicators also gradually increased with increasing BMI (*P*< 0.0001). There was no difference in the TSH, TPOAb, or TGAb values between these six groups.

**Table 2:** Baseline characteristics of study participants.

	Total (n=69007)	Metabolically healthy (35.90%, n= 24774)				Metabolically unhealthy (64.10%, n=44233)				*P* Value
		Normal weight 26.14% (18041)	Overweight 8.00% (5524)	Obese 1.75% (1209)	*P* for trend	Normal weight 25.47% (17574)	Overweight 26.10% (18010)	Obese 12.54% (8649)	*P* for trend	
Age, years	43.53 ± 0.08	36.35 ± 0.14	41.49 ± 0.24	41.27 ± 0.56	<0.0001	45.50 ± 0.19	48.59 ± 0.14	46.11 ± 0.21	<0.0001	<0.0001
Sex(M/F)	34690/34317	6895/11146	2848/2676	658/551	<0.0001	8455/9119	10498/7512	5736/3273	<0.0001	<0.0001
(M%/F%)	(50.27/49.73)	(38.22/61.78)	(51.56/48.44)	(54.41/45.59)	<0.0001	(48.11/51.89)	(58.29/41.71)	(62.16/37.84)	<0.0001	<0.0001
College or above, %	33.49	48.03	35.90	32.20	<0.001	30.30	25.10	24.77	<0.0001	<0.0001
Income≧¥30000/year, %	55.65	59.95	58.29	59.53	<0.0001	52.42	53.39	55.28	<0.0001	<0.0001
Current smoker, %	22.95	15.89	20.69	22.70	<0.0001	23.28	26.95	30.52	<0.0001	<0.0001
Urban Location, %	51.52	57.89	54.55	54.60	0.006	46.79	49.10	49.84	<0.0001	<0.0001
Ethnic Han, %	95.67	93.80	95.04	93.02	0.015	96.24	97.04	96.48	0.015	<0.0001
BMI, kg/m^2^	24.09 ± 0.02	20.82 ± 0.02	25.52 ± 0.02	30.23 ± 0.12	<0.0001	21.66 ± 0.02	25.83 ± 0.01	30.40 ± 0.03	<0.0001	<0.0001
Waist Circumference, cm	83.44 ± 0.06	74.70 ± 0.08	85.72 ± 0.13	95.07 ± 0.34	<0.0001	78.70 ± 0.08	88.52 ± 0.07	97.75 ± 0.12	<0.0001	<0.0001
Systolic BP, mmHg	126.46 ± 0.10	112.73 ± 0.10	116.79 ± 0.16	118.20 ± 0.33	<0.0001	130.08 ± 0.20	134.97 ± 0.18	138.23 ± 0.28	<0.0001	<0.0001
Diastolic BP, mmHg	78.61 ± 0.06	70.82 ± 0.08	73.19 ± 0.13	74.26 ± 0.28	0.007	80.39 ± 0.13	83.27 ± 0.12	86.11 ± 0.18	0.007	<0.0001
Fasting glucose, mmol/L	5.43 ± 0.01	4.92 ± 0.00	5.06 ± 0.01	5.16 ± 0.02	<0.0001	5.53 ± 0.02	5.77 ± 0.02	5.91 ± 0.02	<0.0001	<0.0001
OGTT-2h PG (mmol/L)	6.54 ± 0.01	5.57 ± 0.02	5.96 ± 0.03	6.14 ± 0.06	<0.0001	6.56 ± 0.03	7.26 ± 0.03	7.64 ± 0.05	<0.0001	<0.0001
HbA1c	5.61 ± 0.01	5.30 ± 0.00	5.39 ± 0.01	5.48 ± 0.02	<0.0001	5.63 ± 0.01	5.83 ± 0.01	5.92 ± 0.02	<0.0001	<0.0001
LDL-C, mmol/L	2.84 ± 0.00	2.55 ± 0.01	2.86 ± 002	3.00 ± 0.04	<0.0001	2.79 ± 0.01	3.04 ± 0.01	3.15 ± 0.01	<0.0001	<0.0001
HDL-C, mmol/L	1.46 ± 0.00	1.68 ± 0.00	1.56 ± 0.01	1.55 ± 0.02	<0.0001	1.44 ± 0.00	1.31 ± 0.00	1.26 ± 0.01	<0.0001	<0.0001
UA, mmol/L	316.85 ± 0.49	285.49 ± 0.85	309.96 ± 1.64	328.59 ± 3.84	<0.0001	308.46 ± 1.06	336.34 ± 0.92	362.38 ± 1.42	<0.001	<0.0001
TC, mmol/L	4.80 ± 0.01	4.51 ± 0.01	4.71 ± 0.02	4.75 ± 0.04	<0.001	4.77 ± 0.01	5.02 ± 0.01	5.05 ± 0.02	<0.001	<0.0001
TG, mmol/L	1.59 ± 0.01	0.88 ± 0.00	1.03 ± 0.01	1.12 ± 0.01	<0.001	1.65 ± 0.03	2.11 ± 0.02	2.36 ± 0.03	<0.001	<0.0001
TSH, mIU/L	2.93 ± 0.02	2.79 ± 0.05	2.77 ± 0.04	3.14 ± 0.23	0.214	2.96 ± 0.04	2.98 ± 0.04	3.10 ± 0.07	0.231	<0.0001
TPOAb	34.14 ± 0.46	34.42 ± 1.01	34.69 ± 1.55	33.93 ± 3.52	0.830	35.23 ± 0.92	33.16 ± 0.84	33.12 ± 1.19	0.192	0.600
TgAb	72.98 ± 1.47	74.38 ± 2.64	76.92 ± 6.09	75.89 ± 15.09	0.361	77.20 ± 3.09	69.31 ± 2.79	66.39 ± 3.78	0.051	0.200
UIC(µg/L)	251.34 ± 3.61	260.60 ± 7.84	250.89 ± 9.52	233.23 ± 8.68	0.063	237.30 ± 3.29	257.79 ± 9.81	248.67 ± 7.40	0.072	0.020

Data are presented as mean ± standard error (SE) or percentage.

BMI, body mass index; DBP, diastolic blood pressure; SBP, systolic blood pressure; HbA1c, glycosylated hemoglobin; HDL-C, high- density lipoprotein cholesterol; LDL-C, low-density lipoprotein cholesterol; OGTT, oral glucose tolerance test; PG, plasma glucose; TC, total cholesterol; TG, triglyceride; TgAb, thyroglobulin antibody; TPOAb, thyroid peroxidase antibody; TSH, thyrotropin; UIC, urinary iodine concentration.

### 3.2 Prevalence distributions of thyroid obesity disorders by metabolic phenotype and sex

**Figure 1:**
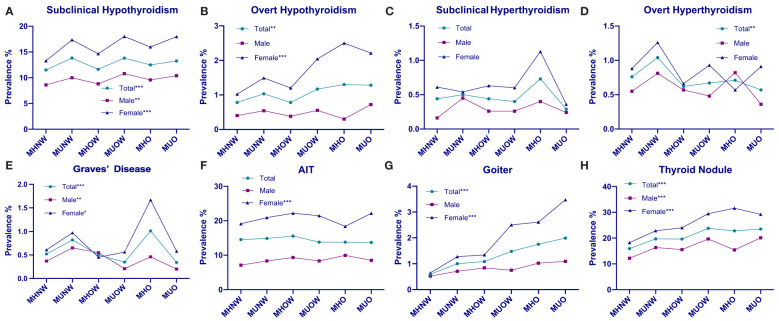
Prevalence distributions of thyroid disorders by metabolic phenotype and sex. **(A)** Prevalence of subclinical hypothyroidism in the overall, male, and female populations, stratified by metabolic obesity phenotype. **(B)** Prevalence of overt hypothyroidism in the overall, male, and female population, stratified by metabolic obesity phenotype. **(C)** Prevalence of subclinical hyperthyroidism in the overall, male, and female population, stratified by metabolic obesity phenotype. **(D)** Prevalence of overt hyperthyroidism in the overall, male, and female population, stratified by metabolic obesity phenotype. **(E)** Prevalence of Graves’ disease in the overall, male, and female population, stratified by metabolic obesity phenotype. **(F)** Prevalence of AIT in the overall, male, and female population, stratified by metabolic obesity phenotype. **(G)** Prevalence of goiter in the overall, male, and female population, stratified by metabolic obesity phenotype. **(H)** Prevalence of thyroid nodule in the ove rall, male, and female population, stratified by metabolic obesity phenotype. *P < 0.05 for trend. **P < 0.01for trend. ***P < 0.001 for trend.

We compared the prevalence of different thyroid disorders based on six metabolic phenotypes for obesity and sex, which is summarized in **Table 3** and **Figure 1**. The prevalence of subclinical hypothyroidism in the overall population was 12.92% [CI 12.58–13.26%], that of overt hypothyroidism was 1.02% [CI 0.92–1.11%], that of subclinical hyperthyroidism was 0.43% [CI 0.37–0.49%], that of overt hyperthyroidism was 0.77% [CI 0.68–0.86%], that of Graves’ disease was 0.54% [CI 0.46–0.61%], that of AIT was 14.38% [CI 14.03–14.74%], that of goiter was 1.16% [CI 1.07–1.26%]and thyroid nodules was 20.29% [CI 19.87–20.71%]. In addition, the prevalence of these thyroid disorders among females was significantly higher than that among males. There was a significant difference in the prevalence of subclinical hypothyroidism, Graves’ disease and thyroid nodules between these six groups both in the total, female and male populations (all *P* < 0.05). The prevalence of overt hypothyroidism and goiter were related to these six phenotypes in the total and female populations (all *P* < 0.05) but were related to none of the obesity metabolic phenotypes among males (*P* > 0.05). There was a significant difference in the prevalence of overt hyperthyroidism in the total population (*P* < 0.05) that was not correlated with the obesity metabolic phenotypes in the male and female subgroups (*P* > 0.05). The prevalence of AIT was not significantly different in either the total or male population (all *P* > 0.05), whereas that in the female population was significant (*P* < 0.05). No significant differences in the prevalence of subclinical hyperthyroidism were observed between these six groups in the total, female and male populations (all *P* > 0.05).

**Table 3:** Prevalence distributions of thyroid disorders by metabolic obesity phenotype and gender.

	MHNW	MUNW	MHOW	MUOW	MHO	MUO	Total	P Value
Subclinical hypothyroidism (%)								
Total	11.50(10.87,12.12)	13.82(13.13,14.51)	11.64(10.54,12.74)	13.80(13.15,14.45)	12.49(10.06,14.92)	13.26(12.36,14.15)	12.92(12.58,13.26)	<0.0001
Male	8.60(7.67,9.52)	9.99(9.11,10.88)	8.80(7.48,10.12)	10.79(10.01,11.58)	9.57(6.42,12.71)	10.39(9.35,11.43)	9.90(9.48,10.33)	0.01
Female	13.29(12.46,14.12)	17.36(16.33,18.39)	14.66(12.89,16.42)	18.00(16.90,19.11)	15.98(12.24,19.72)	17.97(16.34,19.60)	15.97(15.45,16.49)	<0.0001
Overt hypothyroidism (%)								
Total	0.78(0.61,0.95)	1.03(0.84,1.21)	0.78(0.53,1.02)	1.17(0.98,1.36)	1.30(0.63,1.98)	1.28(0.99,1.57)	1.02(0.92, 1.11)	0.004
Male	0.40(0.20,0.60)	0.54(0.35,0.72)	0.38(0.13,0.64)	0.55(0.39,0.72)	0.30(-0.05,0.65)	0.72(0.41,1.03)	0.53(0.43, 0.62)	0.34
Female	1.02(0.77,1.26)	1.49(1.18,1.80)	1.20(0.78,1.62)	2.04(1.65,2.43)	2.50(1.08,3.91)	2.21(1.62,2.79)	1.51(1.35, 1.67)	<0.0001
Subclinical hyperthyroidism (%)								
Total	0.44(0.32,0.57)	0.50(0.37,0.63)	0.44(0.24,0.64)	0.40(0.29,0.51)	0.73(-0.04,1.50)	0.29(0.15,0.43)	0.43(0.37, 0.49)	0.37
Male	0.16(0.05,0.28)	0.45(0.27,0.64)	0.26(0.07,0.44)	0.26(0.14,0.38)	0.40(-0.38,1.18)	0.24(0.09,0.39)	0.29(0.22, 0.35)	0.17
Female	0.61(0.43,0.80)	0.54(0.36,0.73)	0.63(0.26,0.99)	0.60(0.39,0.80)	1.13(-0.27,2.52)	0.36(0.10,0.63)	0.58(0.48, 0.68)	0.62
Overt hyperthyroidism (%)								
Total	0.76(0.58,0.94)	1.04(0.83,1.25)	0.62(0.37,0.86)	0.67(0.51,0.82)	0.71(0.08,1.34)	0.57(0.35,0.78)	0.77(0.68, 0.86)	0.01
Male	0.55(0.31,0.79)	0.81(0.57,1.05)	0.57(0.23,0.92)	0.48(0.31,0.65)	0.82(-0.13,1.78)	0.36(0.17,0.55)	0.57(0.47, 0.67)	0.12
Female	0.88(0.64,1.13)	1.26(0.91,1.60)	0.66(0.32,1.00)	0.93(0.64,1.22)	0.57(-0.22,1.35)	0.91(0.42,1.39)	0.97(0.82, 1.12)	0.23
Graves’ disease (%)								
Total	0.52(0.37,0.68)	0.82(0.62,1.02)	0.50(0.30,0.71)	0.35(0.25,0.46)	1.01(0.21,1.82)	0.34(0.17,0.51)	0.54(0.46, 0.61)	<0.0001
Male	0.37(0.17,0.58)	0.65(0.43,0.87)	0.55(0.22,0.89)	0.21(0.10,0.31)	0.46(-0.18,1.10)	0.20(0.05,0.35)	0.38(0.29, 0.46)	0.002
Female	0.61(0.40,0.83)	0.97(0.65,1.30)	0.45(0.21,0.69)	0.56(0.35,0.77)	1.67(0.08,3.27)	0.58(0.21,0.95)	0.69(0.57, 0.82)	0.03
AIT (%)								
Total	14.53(13.84,15.22)	14.87(14.18,15.56)	15.55(14.28,16.82)	13.79(13.14,14.43)	13.79(10.93,16.64)	13.68(12.76,14.60)	14.38(14.03,14.74)	0.06
Male	7.12(6.28, 7.95)	8.35(7.57, 9.12)	9.30(7.80,10.79)	8.30(7.62, 8.98)	9.96(5.89,14.02)	8.51(7.54, 9.48)	8.22(7.83, 8.61)	0.1
Female	19.11(18.14,20.08)	20.92(19.84,22.01)	22.20(20.17,24.24)	21.46(20.27,22.64)	18.36(14.36,22.36)	22.16(20.37,23.95)	20.62(20.03,21.20)	0.002
Goiter (%)								
Total	0.59(0.46,0.73)	1.00(0.82,1.18)	1.08(0.77,1.40)	1.48(1.27,1.69)	1.75(0.87,2.62)	1.99(1.65,2.33)	1.16(1.07, 1.26)	<0.0001
Male	0.52(0.30,0.74)	0.71(0.51,0.92)	0.84(0.47,1.22)	0.75(0.56,0.93)	1.02(0.01,2.03)	1.09(0.80,1.39)	0.76(0.66, 0.86)	0.07
Female	0.64(0.46,0.81)	1.27(0.99,1.55)	1.34(0.82,1.86)	2.50(2.07,2.94)	2.61(1.11,4.11)	3.47(2.73,4.21)	1.57(1.41, 1.72)	<0.0001
Thyroid Nodule (%)								
Total	15.92(15.16,16.67)	19.72(18.94,20.51)	19.65(18.28,21.02)	23.77(22.97,24.58)	22.81(19.34,26.27)	23.56(22.38,24.74)	20.29(19.87,20.71)	<0.0001
Male	12.17(11.02,13.33)	16.34(15.28,17.40)	15.55(13.81,17.29)	19.68(18.70,20.66)	15.42(10.85,19.99)	20.12(18.67,21.57)	17.01(16.45,17.56)	<0.0001
Female	18.24(17.25,19.22)	22.86(21.73,23.99)	24.02(21.91,26.13)	29.49(28.16,30.83)	31.62(26.47,36.76)	29.21(27.20,31.22)	23.61(22.97,24.24)	<0.0001

MHNW, metabolically healthy normal weight;MUNW, metabolically unhealthy normal weight; MHOW, metabolically healthy overweight; MUOW, metabolically unhealthy overweight; MHO,metabolically healthy obesity; MUO metabolically unhealthy obesity.

### 3.3 Association between different metabolic phenotypes and thyroid disorders

**Figure 2:**
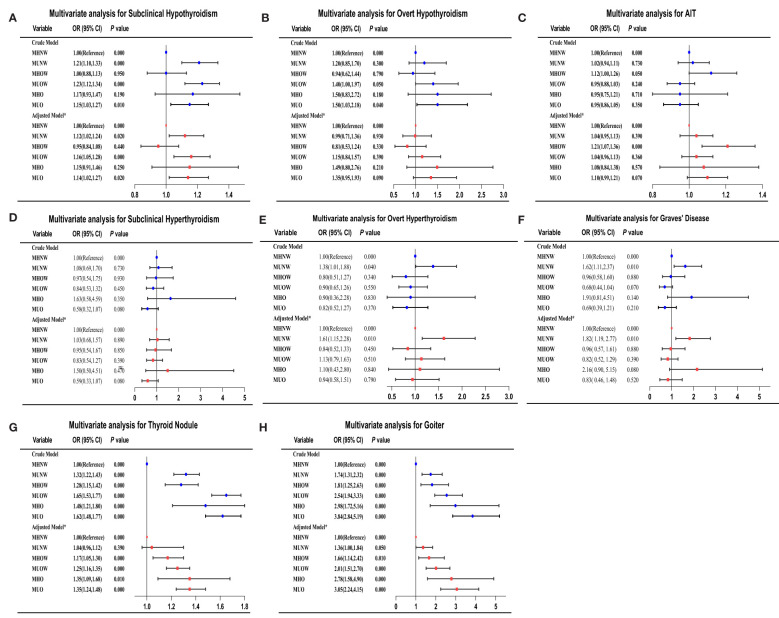
Adjusted ORs and CIs estimating the association between different metabolic obesity phenotypes and thyroid disorders. **(A)** subclinical hypothyroidism; **(B)** overt hypothyroidism; **(C)** AIT; **(D)** subclinical hyperthyroidism; **(E)** overt hyperthyroidism; **(F)** Graves’ disease; **(G)** thyroid nodules; **(H)** goiter. CI, confidence interval; OR, odds ratio. *Multivariate analysis model adjusted for sex, age, smoking status, educational level, family income, UIC, TPOAbs, and TgAbs.

As indicated in **Figure 2** and **Supplementary Table 1**, we analyzed the risk of thyroid

disorders among patients with different metabolic obesity phenotypes. The logistic regression analysis included adjustments for essential demographic, anthropometric, and serological information in the present study. Using the MHNW group as the reference group, the metabolically unhealthy phenotypes were all associated with a higher risk of subclinical hypothyroidism in both crude and adjusted models. Subjects in the MUNW, MUOW, and MUO groups had a 1.13-fold (*P* < 0.01), 1.17-fold (*P* < 0.001) and 1.15-fold (*P* < 0.01) increased risk of subclinical hypothyroidism, respectively, compared to the MHNW phenotype in the fully adjusted model. In the total study population, the association observed between an increased risk of overt hypothyroidism and the MUO phenotype in the unadjusted state disappeared after adjustment for confounding variables, including UIC, TPOAbs and TGAbs. The multiple logistic regression analysis revealed that only the MUNW group was associated with a higher risk of overt hyperthyroidism and Graves’ disease than patients with MHNW (all *P* < 0.01). The associated risks in the fully adjusted model were 1.61 (95% CI = 1.15–2.28) for overt hyperthyroidism and 1.82 (95% CI 1.19-2.77) for Graves’ disease in the MUNW group. Similarly, only the MHOW phenotype was significantly associated with AIT in both the crude and adjusted models. In the fully adjusted model, the ORs for goiter were 1.36 (95% CI, 1.00–1.84) in the MUNW group, 1.66 (95% CI, 1.14–2.42) in the MHOW group, 2.01 (95% CI, 1.51–2.70) in the MUOW group, 2.78 (95% CI, 1.58–4.90) in the MUO group, and 3.05 (95% CI, 2.73–4.13) in the MUO group compared with the MHNW group. Among the subjects with thyroid nodules, the OR for the MHOW group was 1.17 [CI 1.05–1.30], the OR for the MUOW group was 1.25 [CI 1.16–1.35], the OR for the MHO group was 1.35 [CI 1.09–1.68] and the OR for the MUO group was 1.35 [CI 1.24–1.48] after full adjustment. No differences were found in subclinical hyperthyroidism or different metabolic phenotypes in either crude or adjusted models. A similar trend was also observed in overt hypothyroidism in all adjusted models, but we found that the MUOW and MUO groups had a 1.40-fold (*P* < 0.05) and 1.50-fold (*P* < 0.05) increased risk, respectively, compared to the MHNW phenotype in the crude model.

## 4 Discussion

In recent years, obese individuals who do not present metabolic abnormalities and normal weight individuals who present metabolic abnormalities have attracted widespread attention. Some studies have examined the association between different subtypes of obesity and metabolic abnormalities with the risk of various diseases, such as cancer, cardiovascular disease, and chronic kidney disease (18–20). To our knowledge, this is the first study to evaluate the prevalence of thyroid disorders among different metabolic obesity phenotypes and the relationship between the risks of thyroid disorders and metabolic phenotypes in the Chinese adult population using TIDE study data, a nationally representative database. The important results of this study are as follows: (1) the prevalence of various thyroid diseases differed significantly within the six groups by obesity and metabolism. (2) MUNW subjects were associated with a higher risk of overt hyperthyroidism and Graves’ disease incidence than other subjects. (3) Subjects with unhealthy metabolic phenotypes (MUNW, MUOW, and MUO) all had an increased risk of subclinical hypothyroidism regardless of their BMI status. (4) Subjects with the MUO phenotype (the unhealthiest phenotype) were found to have the highest risk of goiter and thyroid nodules compared with other groups.

Recent studies have demonstrated that an estrogen-induced increase occurs in thyroid follicular cell proliferation (21). In Garber et al.’s review, the prevalence of thyroid disorders was shown to be higher among females, and the male-to-female ratio was 1:4 (22), which is consistent with our results. We found that the prevalence of eight thyroid disorders among females was much higher than that among males. Although previous cohort studies have reported an association between thyroid function and different metabolic obesity phenotypes, the role of obesity and metabolic abnormalities in thyroid disorders is still unclear (23). Amouzegar et al. conducted a 9-year follow-up study among 1938 individuals from the Tehran Thyroid Study (TTS). They found that FT4 was positively related to metabolically healthy nonobesity (MHNO) development and negatively related to metabolically healthy obesity (MHO) development, while TSH was positively related to MUNO development (24). The pathophysiology and management of metabolic abnormality of obesity and different thyroid diseases are different. Different thyroid diseases had different pathophysiology could make Mets, but there is no Mets on hyperthyroidism.

Based on our study, we found that metabolic abnormalities and obesity play different roles in various thyroid diseases. There is general agreement that thyroid hormones regulate basal energy expenditure and influence glucose and lipid metabolism (25). The effects of thyroid hormones on the synthesis, mobilization, and degradation of lipids and different aspects of TG-HDL metabolism and apolipoprotein AV (ApoAV) levels could explain lipid abnormalities in thyroid dysfunction, especially hypothyroidism (26, 27). Subclinical hypothyroidism (SCH), which is defined as isolated elevation of TSH levels with free thyroxine (FT4) and free triiodothyronine (FT3) within the normal range, is a relatively common disorder (28). A cross-sectional study conducted by Mahdavi et al. in an Iranian population found no significant association between obesity and the prevalence of subclinical hypothyroidism (29). Shin et al. conducted a cross-sectional study among 6241 euthyroid subjects. They found that individuals in the lowest FT4 quartile had an increased odds ratio of being insulin resistant in both the normal weight and the overweight/obese groups (30). Similar to these studies, our results suggested that subjects with unhealthy metabolic phenotypes (MUNW, MUOW, and MUO) all had an increased risk of SCH, regardless of their BMI status. In our study, we also found that the prevalence of SCH was almost as high in the MUNW group as in the MUO group. No significant differences were observed in the prevalence of subclinical hypothyroidism among the 3 BMI categories. Consistent with our results, a meta-analysis found that BMI was not positively correlated with the prevalence of subclinical hypothyroidism in a meta-regression (P=0.50) (31).

It is commonly noticed in clinical practice that obese individuals often have higher TSH levels (32). Thyroid hormones are involved in body weight regulation through energy consumption. Hyperthyroidism with excess thyroid hormones is often accompanied by weight loss and increased steatolysis and energy expenditure. The latter phenomenon results from a reduced thermodynamic efficiency of the biological machine with increased heat production (33). A recent meta-analysis performed by Song et al. using 22 studies found a positive association between obesity and the risk of hypothyroidism (OR: 1.86; 95% CI: 1.63–2.11, *P* < 0.001). However, no association was found between obesity and hyperthyroidism (34). Our results also suggested that the prevalence of overt hyperthyroidism and Graves’ disease was highest among MUNW subjects. Similarly, we found that MUNW subjects had an approximately 1.6-fold higher risk of overt hyperthyroidism and a 1.8-fold higher risk of Graves’ disease than their metabolically healthy counterparts (MHNW). In addition, no significant relationship was found between the overweight and obesity groups. An adaptation process to increase energy expenditure as normalization of elevated TSH levels in obese subjects has been reported after weight loss (35). In addition, no significant differences were observed in the prevalence of AIT among the 3 BMI categories. A previous study on subjects without thyroid autoimmunity at baseline found no significant association between the baseline abdominal obesity phenotype and the development of TPOAb positivity (36).

Moreover, our results suggested that the prevalence of goiter and thyroid nodules was related to both obesity and metabolic abnormalities. The prevalence of these two diseases increased with higher BMI values, and the MUO group had the highest incidence. The present study also demonstrated that the MUO group had significantly larger thyroid volumes and the highest risk for goiter and thyroid nodules among the obesity metabolic phenotypes. Correlations between BMI, metabolic factors and thyroid nodules have been confirmed by some previous studies. In previous studies, Ayturk et al. (37) and Rezzonico et al. (13) indicated that subjects with MetS had a larger thyroid gland volume by ultrasound and had a significantly increased thyroid nodule prevalence. Demir et al. (38) assessed the antiproliferative and pleiotropic effects of statins on thyroid volume and nodularity. They found that the total thyroid volume decreased more among patients receiving 20 mg of rosuvastatin than in the control group (p < 0.05). Xu et al. (39) investigated the prevalence of thyroid nodules (TNs) and their related factors and found that metabolic factors, including central obesity, hypertension, diabetes and fatty liver, were positively associated with TNs. In most of these studies, thyroid volume was significantly positively correlated with BMI (40). In a recent local community study, Zhu et al. investigated the prevalence of TNs and their related factors and found that being overweight might be a risk factor for TNs (41). Although a causal relationship was not specifically found, the association between thyroid volume and BMI has been asserted.

In our study, we could draw the conclusion that metabolically unhealthy individuals, both with and without obesity, have a higher risk of thyroid disorder incidence than metabolically healthy individuals without obesity. Metabolically healthy individuals exhibit lower thyroid disorder prevalence than their unhealthy counterparts at each BMI level. Furthermore, it has been reported that leptin has direct effects on the formation of thyroid disorders. Leptin promotes thyrotropin-releasing hormone (TRH) expression and synthesis in the paraventricular hypothalamic and arcuate nucleus, which, in turn, can cause an increase in serum TSH levels. Excess leptin secreted from greater amounts of adipose tissue may stimulate the hypothalamic–pituitary–thyroid axis, increasing thyrotropin secretion (42).

Some studies have reported that leptin levels were substantially higher in MUO individuals than in MHO individuals (43). In addition, an inverse correlation between adiponectin and proteinuria was found (44), and adiponectin was higher in individuals with a metabolically healthy phenotype than in metabolically abnormal individuals independent of body mass index (45). This explains why hypothyroidism was more associated with metabolically unhealthy individuals in our results. It has also been reported that the relationship between obesity and thyroid nodules is probably related to leptin secreted by adipose tissue (46). Elevated serum leptin concentrations in obese individuals can promote increased thyroid-stimulating hormone levels, leading to the occurrence and development of thyroid nodules.

Several strengths of our study should be noted, including the large sample size, the population-based design, suitable exclusion criteria, and the performance of all examinations by the same trained staff. However, this study also has some limitations, which should be addressed. First, this was a cross-sectional study, so we could not confirm the cause-and-effect relationship between metabolic obesity phenotypes and thyroid disorders. Second, epidemiological investigations are methodologically challenged by the impact of geographic, environmental, genetic, drug and other factors that may influence disease outcomes, and therefore, reverse causation or confounding should be kept in mind. Third, since our study excluded the usage of all drugs with potential effects on thyroid function and only included adults, obesity and overweight were defined solely based on BMI values. Therefore, it is uncertain whether the findings can be generalized to the entire Chinese population. Finally, the analyses did not include other thyroid-related serum elements, such as free triiodothyronine (FT3) or anti-TSH receptor antibodies.

## 5 Conclusion

In conclusion, this cross-sectional study in a Chinese population demonstrates that metabolically unhealthy individuals have a higher risk of thyroid disorder incidence than metabolically healthy individuals regardless of BMI status. The findings indicate that all metabolically unhealthy individuals, even those without obesity, should be encouraged to improve their metabolic status to reduce the risk of thyroid disorders.

## Author’s note

The authors hereby confirm that neither the manuscript nor any part of it has been published or is being considered for publication elsewhere. We acknowledge that all authors participated sufficiently in the work and take public responsibility for its content.

## Data availability statement

The original contributions presented in the study are included in the article/**Supplementary Material**. Further inquiries can be directed to the corresponding author.

## Ethics statement

The studies involving human participants were reviewed and approved by The Medical Ethics Committee of China Medical University. (2014-103-2; serial number: IRB [2008]115). The patients/participants provided their written informed consent to participate in this study.

## Author contributions

BS is the first author of this study. DT is the corresponding author supervising this work. BS managed the case and drafted the manuscript. CL provided major statistical and technical support. BS, CL, WT and ZS assisted in literature review and organizing data from the literature. WT and ZS reviewed the manuscript. The data of the cross-sectional study is from the TIDE survey group. All authors contributed to the article and approved the submitted version.
