# Supplementary material for: Association between different metabolic phenotypes of obesity and thyroid disorders among Chinese adults: a nationwide cross-sectional study
Source: Front Endocrinol (Lausanne). 2023 Apr 21;14:1158013. doi: 10.3389/fendo.2023.1158013 (PMC10162017; doi:10.3389/fendo.2023.1158013)
Supplement: Supplementary file 1 [file DataSheet_1.docx]

**Supplementary TableS1. The Association between different metabolic phenotypes and thyroid diseases**

|  | MHNW | MUNW | MHOW | MUOW | MHO | MUO |
| --- | --- | --- | --- | --- | --- | --- |
| Subclinical hypothyroidism | | | | | | |
| Crude Model | Reference | 1.21(1.10-1.33) *** | 1.00(0.88-1.13) | 1.23(1.12-1.34) *** | 1.17(0.93-1.47) | 1.15(1.03-1.27) ** |
| Adjusted Model 1 | Reference | 1.11(1.01-1.23) * | 0.97(0.86-1.10) | 1.15(1.04-1.26) * | 1.14(0.91-1.43) | 1.14(1.02-1.27) * |
| Adjusted Model 2 | Reference | 1.13(1.03-1.24) ** | 0.98(0.86-1.11) | 1.17(1.06-1.28) *** | 1.15(0.91-1.45) | 1.15(1.03-1.28) ** |
| Overt hypothyroidism | | | | | | |
| Crude Model | Reference | 1.20(0.85-1.70) | 0.94(0.62-1.44) | 1.40(1.00-1.97) * | 1.50(0.83-2.72) | 1.50(1.03-2.18) * |
| Adjusted Model 1 | Reference | 1.03(0.75-1.42) | 0.91(0.60-1.38) | 1.21(0.89-1.66) | 1.45(0.80-2.61) | 1.43(1.01-2.03) * |
| Adjusted Model 2 | Reference | 0.99(0.71-1.36) | 0.81(0.53-1.24) | 1.15(0.84-1.57) | 1.49(0.80-2.76) | 1.35(0.95-1.93) |
| AIT |  |  |  |  |  |  |
| Crude Model | Reference | 1.02(0.94-1.11) | 1.12(1.00-1.26) * | 0.95(0.88-1.03) | 0.95(0.75-1.21) | 0.95(0.86-1.05) |
| Adjusted Model 1 | Reference | 1.04(0.95-1.12) | 1.20(1.07-1.35) * | 1.04(0.95-1.13) | 1.08(0.84-1.39) | 1.09(0.99-1.21) |
| Adjusted Model 2 | Reference | 1.04(0.95-1.13) | 1.21(1.07-1.36) * | 1.04(0.96-1.13) | 1.08(0.84-1.38) | 1.10(0.99-1.21) |
| Subclinical hyperthyroidism | | | | | | |
| Crude Model | Reference | 1.08(0.69-1.70) | 0.97(0.54-1.75) | 0.84(0.53-1.32) | 1.63(0.58-4.59) | 0.58(0.32,1.07) |
| Adjusted Model 1 | Reference | 1.04(0.68-1.58) | 1.00(0.57-1.76) | 0.83(0.54-1.28) | 1.67(0.60-4.67) | 0.60(0.34-1.08) |
| Adjusted Model 2 | Reference | 1.03(0.68-1.57) | 0.95(0.54-1.67) | 0.83(0.54-1.27) | 1.50(0.50-4.51) | 0.59(0.33-1.07) |
| Overt hyperthyroidism | | | | | | |
| Crude Model | Reference | 1.38(1.01-1.88) * | 0.80(0.51-1.27) | 0.90(0.65-1.26) | 0.90(0.36-2.28) | 0.82(0.52-1.27) |
| Adjusted Model 1 | Reference | 1.59(1.14-2.22) ** | 0.93(0.58-1.47) | 1.16(0.81-1.66) | 1.06(0.42-2.67) | 0.95(0.60-1.52) |
| Adjusted Model 2 | Reference | 1.61(1.15-2.28) ** | 0.84(0.52-1.33) | 1.13(0.79-1.63) | 1.10(0.43-2.80) | 0.94(0.58-1.51) |
| Graves’ disease | | | | | | |
| Crude Model | Reference | 1.62(1.11-2.37) ** | 0.96(0.58-1.60) | 0.68(0.44-1.04) | 1.91(0.81-2.67) | 0.69(0.39-1.21) |
| Adjusted Model 1 | Reference | 1.77(1.19-2.65) ** | 1.07(0.64-1.77) | 0.81(0.52-1.27) | 2.17(0.92-5.12) | 0.84(0.47-1.48) |
| Adjusted Model 2 | Reference | 1.82(1.19-2.77) ** | 0.96(0.57-1.61) | 0.82(0.52-1.29) | 2.16(0.90-5.15) | 0.83(0.46-1.48) |
| Goiter | | | | | | |
| Crude Model | Reference | 1.74(1.31-2.32) *** | 1.81(1.25-2.63) * | 2.54(1.94-3.33) *** | 2.98(1.72-5.16) *** | 3.05(2.73-5.19) *** |
| Adjusted Model 1 | Reference | 1.41(1.04-1.91) * | 1.74(1.20-2.54) * | 2.08(1.55-2.77) *** | 2.89(1.66-5.02) *** | 3.14(2.32-4.27) *** |
| Adjusted Model 2 | Reference | 1.36(1.00-1.84) * | 1.66(1.14-2.42) ** | 2.01(1.51-2.70) *** | 2.78(1.58-4.90) *** | 3.05(2.73-4.13) *** |
| Thyroid Nodule | | | | | | |
| Crude Model | Reference | 1.32(1.22-1.43) *** | 1.28(1.15-1.42) *** | 1.65(1.53-1.77) *** | 1.48(1.21-1.80) *** | 1.62(1.48-1.77) *** |
| Adjusted Model 1 | Reference | 1.05(0.97-1.14) | 1.16(1.04-1.28) ** | 1.25(1.16-1.35) *** | 1.36(1.10-1.68) ** | 1.35(1.24-1.48) *** |
| Adjusted Model 2 | Reference | 1.04(0.96-1.12) | 1.17(1.05-1.30) * | 1.25(1.16-1.35) *** | 1.35(1.09-1.68) ** | 1.35(1.24-1.48) *** |

Crude Model: Adjusted for age and sex; Adjusted Model 1: Adjusted for age, sex and ethnicity; *Adjusted model 2: Adjusted for sex, age, smoking status, educational level, family income, UIC, TPOAbs, and TgAbs. *P < 0.05 for trend. **P < 0.01for trend. ***P < 0.001 for trend.

**Supplementary TableS2. The prevalence of thyroid disorders according to different BMI status and metabolic status**

|  | Subclinical hypothyroidism (%) | Overt hypothyroidism (%) | Subclinical hyperthyroidism (%) | Overt hyperthyroidism (%) |
| --- | --- | --- | --- | --- |
| Total | 12.92(12.58-13.26) | 1.02(0.92-1.11) | 0.43(0.37-0.49) | 0.77(0.68-0.86) |
| BMI Status | | | | |
| Normal Weight | 12.61(12.15-13.07) | 0.90(0.78-1.03) | 0.47(0.38-0.56) | 0.89(0.75-1.03) |
| Overweight | 13.29(12.72-13.85) | 1.08(0.92-1.24) | 0.41(0.31-0.51) | 0.65(0.52-0.79) |
| Obese | 13.17(12.33-14.01) | 1.28(1.01-1.55) | 0.34(0.19-0.49) | 0.58(0.38-0.79) |
| P Value | 0.15 | 0.01 | 0.34 | 0.01 |
| Metabolic Status | | | | |
| Metabolic Healthy | 12.16(11.74-12.58) | 0.76(0.66-0.87) | 0.41(0.33-0.49) | 0.86(0.74-0.99) |
| Metabolic Unhealthy | 14.09(13.56-14.62) | 1.41(1.24-1.58) | 0.46(0.36-0.57) | 0.62(0.50-0.75) |
| P Value | <0.0001 | <0.0001 | 0.4 | 0.01 |
|  | Graves’ disease (%) | AIT (%) | Goiter (%) | Thyroid Nodule (%) |
| Total | 0.54(0.46-0.61) | 14.38(14.03-14.74) | 1.16(1.07-1.26) | 20.19(19.87-20.71) |
| BMI Status | | | | |
| Normal Weight | 0.66(0.54-0.79) | 14.69(14.20-15.18) | 0.79(0.68-0.90) | 17.74(17.20-18.29) |
| Overweight | 0.39(0.29-0.49) | 14.21(13.63-14.78) | 1.39(1.21-1.56) | 22.80(22.10-23.49) |
| Obese | 0.42(0.24-0.60) | 13.69(12.81-14.57) | 1.96(1.65-2.28) | 23.47(22.35-24.59) |
| P Value | 0.002 | 0.12 | <0.0001 | <0.0001 |
| Metabolic Status | | | | |
| Metabolic Healthy | 0.63(0.52-0.74) | 13.49(13.05-13.92) | 0.74(0.64-0.83) | 17.42(16.92-17.91) |
| Metabolic Unhealthy | 0.38(0.29-0.48) | 15.75(15.19-16.31) | 1.81(1.62-2.00) | 24.68(24.02-25.355) |
| P Value | <0.001 | <0.0001 | <0.0001 | <0.0001 |

**Supplementary FigureS1. Diagram of study patient selection and stratification.**


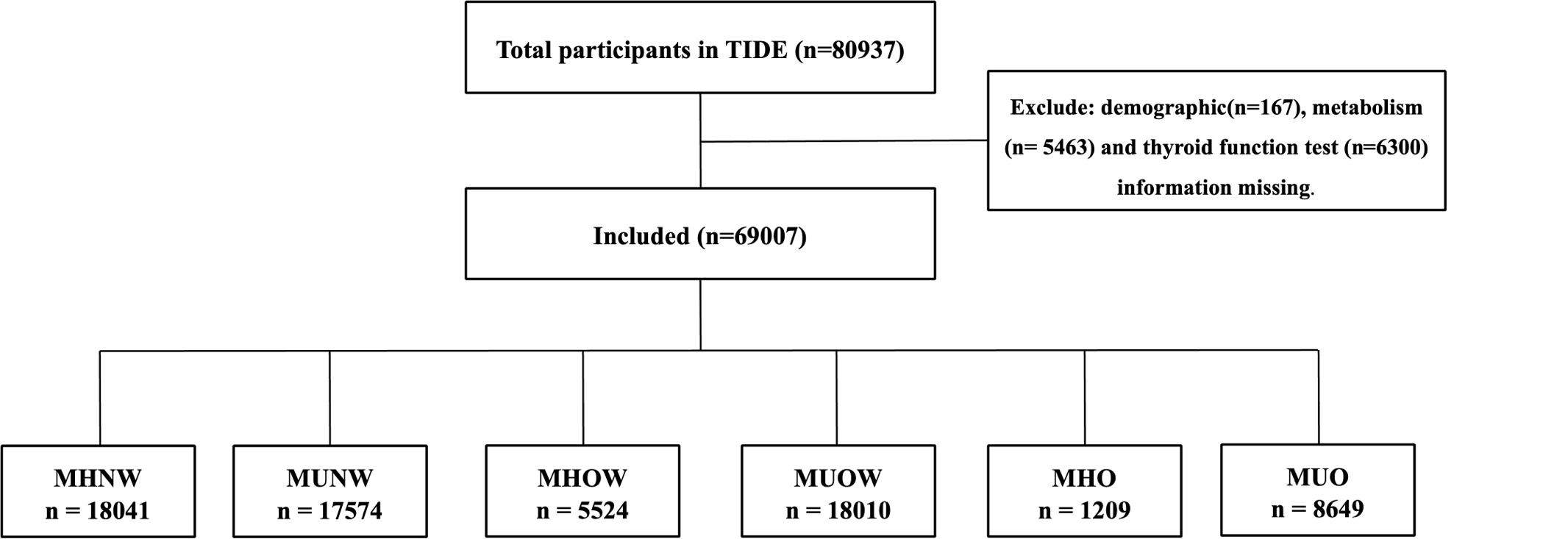


Abbreviations: metabolically healthy normal weight (MHNW); metabolically unhealthy normal weight (MUNW); metabolically healthy overweight (MHOW); metabolically unhealthy overweight (MUOW); metabolically healthy obesity (MHO); metabolically unhealthy obesity (MUO)

**Supplementary Method 1**

The entire study advanced through four stages of random sampling from urban and rural locations in parallel. At the first stage, thirty-one cities were selected and divided into 10 developed, 13 developing, and 8 underdeveloped cities, based on population size and economical levels. At the second stage, one district was randomly selected from each city. At the third stage, two residential districts were randomly selected in a municipal district according to inclusion criteria of age and sex. And at the final stage, eligible individuals who met the inclusion criteria were randomly selected and stratified by age and sex. The age and sex composition of each community and urban-rural ratio were decided referring to the 2010 China’s national census data. A parallel random sampling was performed in rural locations. The inclusion criteria were as follow: aged 18 years or older; Having resided locally for at least 5 years; no iodine-containing drugs or contrast agents within 3 months of participation; non-pregnant women.

**Supplementary Method 2**

**Thyroid disorder incidence according to metabolic health and BMI status**

As shown in Supplementary TableS2, the incidence of these thyroid disorders in the total population was compared based on different metabolic and BMI statuses. We found that the prevalence of overt hypothyroidism, goiter and thyroid nodules was significantly related to both metabolic status and BMI status (all *P* < 0.05), and with increasing BMI, the prevalence increased. For instance, there was a typical trend in the prevalence of goiter between these six groups, with only 0.59% of subjects in the MHNW group having goiter compared to 1.00% in the MUNW group, 1.08% in the MHOW group, 1.48% in the MUOW group, 1.75% in the MHO group and 1.99% in the MUO group, which was three times as high as in the MHNW group. In addition, individuals with an unhealthy metabolic status had a significantly higher prevalence of the above diseases than metabolically healthy participants (all *P* < 0.05). Among them, the prevalence of goiter and overt hypothyroidism in the metabolically unhealthy group was nearly twice as high as that in the healthy group. Similar trends were also observed in the prevalence of subclinical hypothyroidism and AIT between the metabolically unhealthy and healthy groups (all *P* < 0.05). Moreover, we found that the prevalence of these two disorders was associated with metabolic status rather than BMI status (all *P* > 0.05). No statistically significant differences in the prevalence of subclinical hyperthyroidism were found in either the BMI group or the metabolic group (all *P* > 0.05). There were significant differences in the prevalence of overt hyperthyroidism and Graves’ disease in both the BMI group and the metabolic group. However, we found that the prevalence of these two diseases was highest in the normal weight and metabolically healthy groups.
